# Body-Size Change in a Rodent Is Affected by Environmental Warming and Population-Specific Thermoneutral Zone

**DOI:** 10.3390/ani15081112

**Published:** 2025-04-11

**Authors:** Yan Chen, Ke Li, Stefan Sommer, Arpat Ozgul, Yizhen Zhang, Deng Wang

**Affiliations:** 1 College of Grassland Science and Technology, China Agricultural University, 2 Yuanmingyuan West Road, Haidian District, Beijing 100193, China; chenyan01@cau.edu.cn (Y.C.); zhangyizhen@cau.edu.cn (Y.Z.); 2 College of Life Sciences, Henan Normal University, 46 Jianshe East Road, Muye District, Xinxiang 453007, China; 2023245@htu.edu.cn; 3 Department of Evolutionary Biology and Environmental Studies, University of Zurich, Winterthurerstrasse 190, CH-8057 Zurich, Switzerland; stefan.sommer@ieu.uzh.ch (S.S.); arpat.ozgul@uzh.ch (A.O.)

**Keywords:** body size, climate warming, temporal variation, thermoneutral zone, rodent, spatial variation

## Abstract

Rodent body size often exhibits a diversity of temperature–size patterns among and within species, which might be caused by differential thermoneutral zones (TNZs) and experienced extents of warming. Here, we test this hypothesis in populations of *Apodemus agrarius*. To study how body size varies across space, we analysed data on body size and temperature (specifically, annual mean minimum temperature) from nine sites spanning 1150 m of elevation. Using indirect calorimetry, we also measured the resting metabolic rate at different temperatures to infer the population-specific TNZ. To study how body size changes over time (2013–2020), we analysed body-size data of southern and northern populations from warm and cold sites, respectively. With increasing temperatures across space, body size increased and the TNZ narrowed. Moreover, during the eight years, temperature remained stable at the warm site but rose at the cold site. As a result, body size increased in the population at the cold site but remained stable in the population at the warm site. Finally, the rate of change in body size per 1 °C change in temperature was larger along the temporal than the spatial temperature gradient. Together, these results support our hypothesis that, among rodent populations, differential changes in body size can be caused by site-specific habitat warming and the population-specific TNZ. A population with a narrow TNZ can be restricted in its body-size response to habitat warming.

## Simple Summary

This study investigates how spatial and temporal changes in body size in the rodent Apodemus agrarius are caused by changes in ambient temperature. Contrary to the classical Bergmann rule, which predicts larger body sizes in colder climates, we found that A. agrarius grows larger as temperatures rise. We show that the extent of body-size change is strongly regulated by the magnitude of changes in ambient temperature and the population-specific thermoneutral zone. Our results show that populations in cold areas are more vulnerable to global warming than populations in warm areas. This finding may inform region-specific management strategies for species in the context of global climate change.

## 1. Introduction

Bergmann’s rule is a classical ecogeographic prediction of how body size in endotherms changes with changing environmental temperature [1]. The mechanistic explanation of this rule is heat regulation, which depends on an organism’s ratio of body surface to body volume. This ratio increases with body size, enabling large organisms to retain body heat better under low temperatures at high latitudes [2]. However, many animal species, particularly rodents, exhibit a diversity of temperature–size patterns [3]. In three rodent species in China, for example, both spatial and temporal body-length patterns deviated from the prediction of Bergmann’s rule [4]. These deviations indicate that heat dissipation is unlikely the sole mechanism underlying body-size responses to temperature variation [5,6]. Understanding these deviations is crucial for predicting how species may adapt to ongoing climate change, as shifts in body size can have significant ecological and evolutionary consequences for population survival and ecosystem dynamics.

To explain the diversity of temperature–size patterns in endotherms, various alternative hypotheses to Bergmann’s rule have been proposed, such as the heat mortality hypothesis [7], the resource availability hypothesis [8], and the starvation resistance hypothesis [9]. However, none of these hypotheses can be generalised [10]. A further explanation with some support from field studies invokes thermoregulation-related adaptive responses [11,12]. Li et al. [4], for example, hypothesised that the above-mentioned diversity of temperature–size patterns in three rodent species in China may have been caused by differing patterns of energy allocation among biological processes. Depending on the distinct distribution ranges of the three rodent species and the temperature differences in the species’ ancestral sites, Li et al. [4] further hypothesised that these patterns may have been influenced by both species-specific and population-specific thermoneutral zones (TNZs, the temperature ranges within which no additional energy is required to maintain homeostasis in endotherms) [13] and by the populations’ experienced extents of habitat warming. While this hypothesis can account for the diversity of temporal and spatial temperature–size patterns in endotherms, it also aligns with the classical evolutionary principle of the “survival of the fittest” [14]. However, despite the explanatory potential of this hypothesis for the patterns observed in the three rodent species, the relationships among environmental warming, the TNZ, and variations in body size still need to be tested against data from different populations within a species.

How body size responds to environmental warming depends on how animals allocate energy to maintenance (survival), growth, reproduction, and various other body functions. For endotherms to maintain optimal conditions for body functions, energy homeostasis—the balancing of food intake and energy expenditure—is essential [15]. Under stable environmental conditions, the energy expended on each life process is expected to remain stable throughout an individual’s life [16]. However, energy expenditure is inherently a dynamic equilibrium regulated by complex feedback pathways involving both intrinsic physiological traits and extrinsic environmental factors [17,18]. These regulatory pathways also affect phenotypic traits such as body size [19]. This redistribution of energy can influence not only individual fitness but also population-level outcomes, potentially affecting reproductive success, survival, and, consequently, long-term population dynamics under changing environmental conditions. Although the life processes regulating body-size responses to climate warming are not yet fully understood [20] and energy-allocation patterns in endotherms are rarely quantified, several studies suggest that ambient temperature as well as species- and population-specific TNZs play key roles in determining how total energy is allocated to the various life processes [21,22,23].

In endotherms, the associations among ambient temperature, energy allocation, and body size have been documented extensively. For example, in the white-plumed honeyeater (*Ptilotula penicillatus*), females changed their energy-allocation pattern when temperature rose during the breeding season, which led to larger offspring and, eventually, larger body sizes in the entire population [24]. Similarly, in the guinea pig *Cavia porcellus*, females in a cold environment (5 °C) allocated more energy to self-maintenance than females in a warm environment (15 °C), which allocated more energy to growth, development, and reproduction, leading to faster body growth and larger individuals [25]. And in the parti-coloured bat *Vespertilio murinus*, males seasonally exposed to 25 °C matured faster than males constantly kept at 10 °C, suggesting that the males in the cold environment shifted energy from sexual maturation to self-maintenance [26]. Moreover, in birds and mammals, metabolic critical temperature appears to be negatively associated with the basal metabolic rate [27], suggesting that energy-allocation patterns in organisms with different TNZs might be affected differently by the same change in ambient temperature. Notably, different species in the same areas tend to have similar TNZs [28], but populations of the same species in different areas (geographical populations) may have different TNZs [29]. This variation in TNZ across populations may be driven by a combination of physiological plasticity and genetic adaptation, both of which enable populations to cope with different thermal environments. For example, a semi-natural population of the Chinese striped hamster (*Cricetulus barabensis*) gradually broadened its TNZ when the ambient temperature gradually decreased [30,31]. However, it is still unclear whether geographical populations in different thermal environments have different TNZs and, if so, whether these population-specific TNZs are linked to spatial and temporal changes in body size caused by changes in ambient temperature.

Spatial and temporal body-size patterns in rodents often deviate from Bergmann’s prediction by exhibiting a diversity of temperature–size patterns. This diversity might be caused by a combination of differential thermoneutral zones (TNZs) among or within species and experienced extents of warming across space and time. Here, we aim to test this hypothesis against spatial and temporal data in Chinese populations of the striped field mouse (*Apodemus agrarius*), a typical cold-adapted species [32]. In mainland China, *A. agrarius* is found exclusively north of 26° N [33]. As we reported previously [4], the body length of this species increased with increasing ambient minimum temperature across time (two decades) and space (20° of latitude). To explain these trends, we hypothesised that geographical populations evolved different TNZs along thermal gradients [4]. Furthermore, we predicted that populations in warm areas may need more warming (i.e., more time) for the body size to change than populations in cold areas. To test this hypothesis and prediction, we analysed temporal and spatial data on the body size of *A. agrarius*, along with data on ambient temperature. The temporal data were collected from 2013 to 2020 in southern and northern China at two sites with markedly different temperatures. The spatial data were collected in 2022 and 2023 at nine sites spanning 1150 m of elevation. In a subset of the caught individuals, we measured the resting metabolic rate to infer population-specific TNZs. Using these data, we (1) quantify temporal and spatial trends in body size of *A. agrarius*, (2) estimate the effect of temperature variation on temporal and spatial body-size variation, (3) evaluate the effect of temperature on the TNZ, (4) assess the relationship between the TNZ and body size across space, and (5) test the effect of the TNZ on body-size changes over time. Our results support the hypothesis and confirm the prediction. As such, they improve our understanding of how changes in habitat temperature and thermos physiology interact to explain the diverse temperature–size patterns in rodents. Importantly, our study fills a critical gap in understanding how population-specific TNZs may be linked to body-size variation across both spatial and temporal scales, offering new insights into the interplay between climate warming and species’ physiological responses.

## 2. Materials and Methods

### 2.1. Rodent Data

We used spatial and temporal data. The spatial data were collected from June to August in 2022 and 2023. In these years, 180 *A. agrarius* individuals were captured with live traps at nine sites in Guizhou Province along an elevation gradient of about 1150 m (Figure S1). Of the individuals captured in 2023, 57 adults were used to measure resting metabolic rate (RMR). The temporal data were originally collected for a rodent monitoring programme. During this programme, monthly snap trapping was conducted from 2013 to 2020 in one or two stationary 10-hectare fields at two sites, separated by about 16.5° of latitude (Figure S1). At the southern site in Yuqing (Guizhou Province), 1845 *A. agrarius* individuals were captured; at the northern site in Gongzhuling (Jilin Province), 869 *A. agrarius* individuals were captured. All captured individuals were sexed and dissected, and their body mass, body length (except for individuals captured at Gongzhuling from 2015 to 2017), and reproductive condition were recorded. Sex was determined by external genital morphology, and pregnancy status was first assessed by dissection, then confirmed by inspection of uterine horns for embryos. An electronic scale was used to weigh each rodent to the nearest 0.01 g. Moreover, based on body-mass criteria [34], each individual was classified into one of five stages: juvenile (less than 16 g), sub-adult (more than 16 g but maximally 23 g), adult I (more than 16 g but maximally 29 g), adult II (more than 29 g but maximally 37 g), or old (more than 37 g). However, because neither juvenile nor old individuals were caught in some years and at some sites, we excluded these two stages from the temporal and spatial modelling of body length and body mass. We also excluded pregnant females because pregnancy status affects body mass. These exclusions were necessary to ensure that the data represent comparable stages of development and non-confounded body mass measurements. As a result of these exclusions, the spatial body-size analyses in Guizhou Province comprised 163 individuals for both length and mass (Table S1). The temporal body-size analysis in Yuqing comprised 1588 individuals for length and mass, and in Gongzhuling, it comprised 307 individuals for length and 552 individuals for mass (Table S2).

### 2.2. Measuring Resting Metabolic Rate (RMR) and Estimating Thermoneutral Zone (TNZ)

Wild-caught animals from the nine geographical populations in Guizhou Province (Figure S1, Table S1) were housed individually in plastic cages (L × W × H, 290 mm × 178 mm × 160 mm) in a laboratory in Yuqing County. Each animal was acclimated for two days before it was used in a trial. Two hours before the trial began, we removed food from the cage. Before the trial began, we measured the animal’s body weight. Then, we placed the animal into a metabolic chamber (volume, 2 L) and measured its oxygen consumption at distinct temperatures (one per trial) using indirect calorimetry [35]. Following Zhao et al. [30] and considering that the RMR increases faster with rising temperature in cold than in warm environments [36], we measured oxygen consumption first at every 2.5 °C from 37.5 °C down to 25 °C, then every 5 °C down to 5 °C. At each temperature, oxygen consumption was recorded automatically at one-minute intervals with an open-flow respirometry system (SANS Biotechnology Co., Ltd.; Beijing, China). Temperature-specific RMR was measured as the minimal oxygen consumption during at least 5 min. Then, this value was converted to the mass-specific oxygen consumption rate (mL O_2_ g^−1^ h^−1^) [30]. Finally, using the gradient descent algorithm [37], we determined the functional relationship between mass-specific RMR and temperature and derived from this relationship the lower limit temperature (LLT), the upper limit temperature (ULT), and the breadth of the TNZ (TNZ_b_ = ULT − LLT). Details on these procedures are provided in Appendix A.

### 2.3. Weather Data

In a previous study [4], the critical climate variable affecting body size in *A. agrarius* was found to be the annual mean minimum temperature in the year of capture (AnnMinTemp). Here, to investigate the effects of spatial and temporal variation in AnnMinTemp on body size and physiological traits in *A. agrarius*, we extracted data on daily minimum temperature from the ERA5-land dataset. The dataset is available at a spatial resolution of approximately 11 km × 11 km on the website of the European Centre for Medium-Range Weather Forecasts (ECMWF, https://cds-beta.climate.copernicus.eu/; accessed on 20 February 2024). For the temporal analysis, we calculated AnnMinTemp per sampling site and year. For the spatial analysis, we calculated AnnMinTemp per sampling site as the mean value of 2022 and 2023.

### 2.4. Data Analysis

To estimate temporal and spatial trends in body size and to study the effects of AnnMinTemp and TNZ_b_ on body size across space, we built generalised linear mixed-effects models (GLMMs) with gamma distributions and an identity link [38]. We used gamma distributions because body-size data, particularly in ecological datasets, tend to be positively skewed. All models included either body length or body mass (of individual *i*) as the response variable (*y_i_*). The trend models included the variables *latitude*, *longitude*, or *altitude* (Equation (1)), and *year* (Equation (2)) as continuous predictors. The effect models included the variables *AnnMinTemp* or *TNZ_b_* as continuous predictors (Equation (3)). Moreover, all models included *sex* and *stage* as fixed effects. To account for random variation across years or sites, we also included *year* (in the temporal analysis) or *site* (in the spatial analysis) as a random effect.

(1) $$y_{i}=\alpha +Latitude\;or\;Longitude\;or\;Altitude+{Sex}_{i}+{Stage}_{i}+Site+\varepsilon$$

(2) $$y_{i}=\alpha +cYear+{Sex}_{i}+{Stage}_{i}+Year+\varepsilon$$

(3) $$y_{i}=\alpha +AnnMinTemp\;or\;TNZ_{b}+{Sex}_{i}+{Stage}_{i}+Site\;or\;Year+\varepsilon$$

The *cYear* variable (Equation (2)) expresses years passed since the beginning of the study period (i.e., year 2013 becomes *cYear* 0, year 2014 becomes *cYear* 1, etc.); *α* and *ε* represent intercept and random error, respectively. To estimate temporal and spatial trends in AnnMinTemp and to study the effects of AnnMinTemp on the ULT, LLT, and TNZ_b_ of populations across space, we constructed linear models (LMs). The trend models (regression models) included AnnMinTemp as the response variable and latitude, longitude, altitude, and year as continuous predictors. The effect models included either ULT, LLT, or TNZ_b_ as the response variable and AnnMinTemp as a continuous predictor.

Finally, we built structural equation models (SEMs) to assess the importance of direct effects of AnnMinTemp on body length and TNZ_b_ and to determine whether the effects between body length and TNZ_b_ were uni- or bidirectional. In the SEMs, we only included variables that had significant effects in the GLMMs and the LMs. For the nine populations of the spatial dataset, the initial SEM included the variables of AnnMinTemp, body length, and TNZ_b_. With these variables, we analysed three pathways of increasing complexity. In the first pathway, we included only the direct effect of AnnMinTemp on body length and TNZ_b_; in the second pathway, we also included the direct effect of TNZ_b_ on body length; and in the third pathway, we further included the bidirectional effect between TNZ_b_ and body length. In all SEMs, the effects of AnnMinTemp or TNZ_b_ on body length were fit as GLMMs, and the effect of AnnMinTemp on TNZ_b_ and the bidirectional effect between TNZ_b_ and body length were fit as LMs. For each GLMM and LM in the SEMs, the response variables, the predictors, and the fixed (Sex and Stage) and random effects (Sites) were the same as the corresponding pair of variables in the GLMMs and LMs in the effect models above. The SEMs were implemented with maximum-likelihood estimation procedures and ranked by AIC values [39]. Model fit was evaluated using Fisher’s C test [40].

For all analyses, we standardised each continuous predictor variable (latitude, longitude, altitude, AnnMinTemp, and TNZ_b_) by subtracting the variable’s mean from individual data points and dividing the difference by the variable’s standard deviation. We conducted all analyses in R version 4.0 [41]. The polynomial regression model was constructed using the nls2 (version 0.3) [42], stats (version 4.0.3) [41], and nleqslv (version 3.3.5) packages [43]. The partial derivatives of the model and the predictions of the TNZ values were calculated using the numDeriv package (version 2016.8) [44]. The GLMMs and LMs were fit using the lme4 (version 1.1) [45] and MASS (version 7.3) [46] packages, respectively. AIC-based model selection was conducted using the AICcmodavg package (version 2.3) [47], and the SEMs were constructed using the piecewiseSEM package (version 2.1.2) [48].

## 3. Results

### 3.1. Spatial and Temporal Trends in Temperature and Body Size

**Figure 1:**
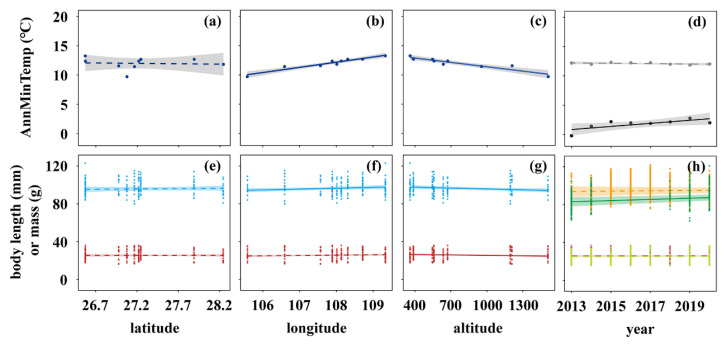
Temperature and body-size trends over time and across space. Top row: Dark-blue lines show changes in annual mean minimum temperature (AnnMinTemp) across nine county sites at different (**a**) latitudes, (**b**) longitudes, and (**c**) altitudes. The lines in (**d**) show temporal temperature trends at the southern site in Yuqing (grey line) and at the northern site in Gongzhuling (black line). Bottom row: Light-blue lines (body length) and red lines (body mass) show changes in the body size of *A. agrarius* across nine county sites at different (**e**) latitudes, (**f**) longitudes, and (**g**) altitudes. The lines in (**h**) show temporal body-size trends in Yuqing (orange line: body length; yellow line: body mass) and Gongzhuling (green line: body length; purple line: body mass). Note that the two lines for body mass overlap. Temperature and body-size lines were fit using LMs and GLMMs, respectively. Solid and dashed lines indicate significant (*p* < 0.05) and non-significant (*p* > 0.05) trends, respectively; shaded areas are the 95% confidence intervals.

Within the study area, the annual mean minimum temperature (AnnMinTemp) did not change significantly across latitudes (*p* > 0.05, Figure 1a), but it increased significantly with increasing longitude (0.89 ± 0.10 °C °E^−1^, estimate ± s.e., *p* < 0.05, Figure 1b). It also decreased significantly with increasing altitude (−2.32 ± 0.37 °C km^−1^, *p* < 0.05, Figure 1c). Over the study period, AnnMinTemp rose significantly at the northern site in Gongzhuling (0.26 ± 0.10 °C yr^−1^, *p* < 0.05, Figure 1d) but remained stable at the southern site in Yuqing (*p* > 0.05, Figure 1d).

Body size of *A. agrarius* exhibited various temporal and spatial patterns. Across latitudes, body length and mass both remained stable (both *p* > 0.05, Figure 1e). With increasing longitude, however, body length increased significantly (0.98 ± 0.46 mm °E^−1^, *p* < 0.05), whereas body mass remained stable (*p* > 0.05) (Figure 1f). Moreover, with increasing altitude, body length and mass both decreased significantly (body length: −1.02 ± 0.43 mm km^−1^; body mass: −0.50 ± 0.15 g km^−1^; both *p* < 0.05, Figure 1g). Over the study period in Yuqing, body length and mass both remained stable (both *p* > 0.05, Figure 1h); in Gongzhuling, body length increased significantly (1.28 ± 0.35 mm yr^−1^, *p* < 0.05), whereas body mass remained stable (*p* > 0.05, Figure 1h).

### 3.2. Spatial and Temporal Trends of Body Size Along Temperature Gradients

**Figure 2:**
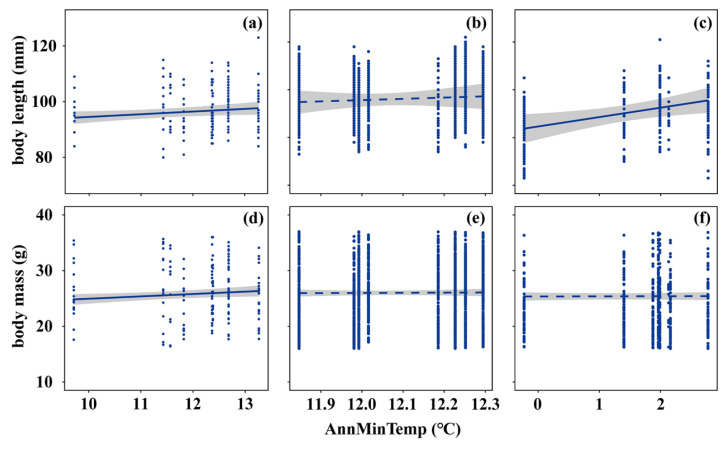
Body-size trends in *A. agrarius* along spatial and temporal gradients of annual mean minimum temperature (AnnMinTemp). Body length (top row) and body mass (bottom row) are corrected for stage and sex across the nine study sites (**a**,**d**) and over the study period (2013–2020) at the southern site in Yuqing (**b**,**e**) and at the northern site in Gongzhuling (**c**,**f**). Note that two sites in the spatial analysis (**a**,**d**) had an AnnMinTemp of about 12.4 °C, and two sites had an AnnMinTemp of about 12.7 °C. Lines were fit using GLMMs; other conventions are the same as in Figure 1.

Across space, the body size of *A. agrarius* increased significantly with increasing AnnMinTemp (body length: 0.99 ± 0.46 mm °C^−1^, *p* < 0.05, Figure 2a; body mass: 0.44 ± 0.19 g °C^−1^, *p* < 0.05, Figure 2d). Over time at the southern site in Yuqing, body length and body mass both remained stable (both *p* > 0.05, Figure 2b,e); at the northern site in Gongzhuling, body length increased significantly with rising AnnMinTemp (3.43 ± 1.30 mm °C^−1^, *p* < 0.05, Figure 2c), while body mass remained stable (*p* > 0.05, Figure 2f).

### 3.3. Interrelations Among Ambient Temperature, Thermoneutral Zone, and Body Size

**Figure 3:**
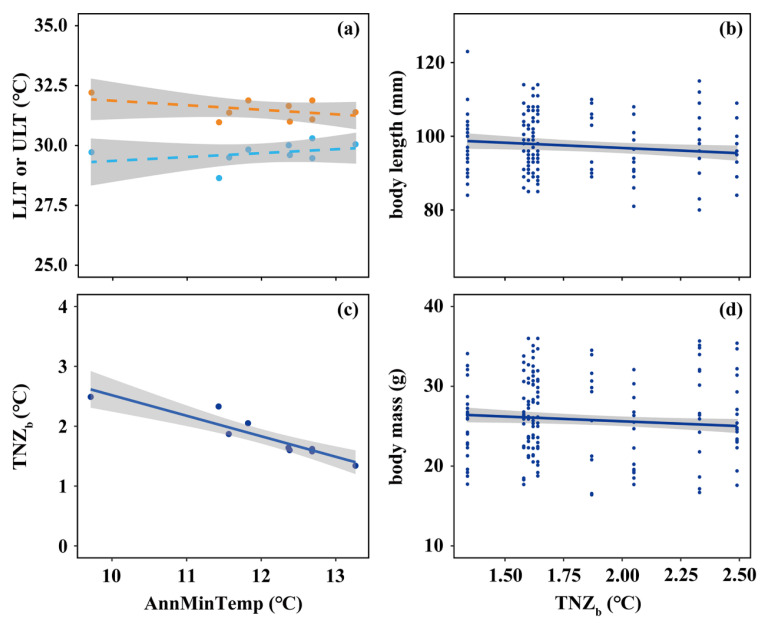
Relationships between characteristics of the thermoneutral zone (TNZ) of *A. agrarius* and annual mean minimum temperature (AnnMinTemp) and between the body size of *A. agrarius* and the breadth of the TNZ (TNZ_b_). Left panels: Trends of (**a**) the upper-limit temperature (ULT, orange), the lower-limit temperature (LLT, light blue), and (**c**) the TNZ_b_ along the spatial temperature gradient. Right panels: Trends of (**b**) the body length and (**d**) body mass of *A. agrarius* along a changing TNZ_b_. Lines were fit using LMs; other conventions are the same as in Figure 1.

**Figure A1:**
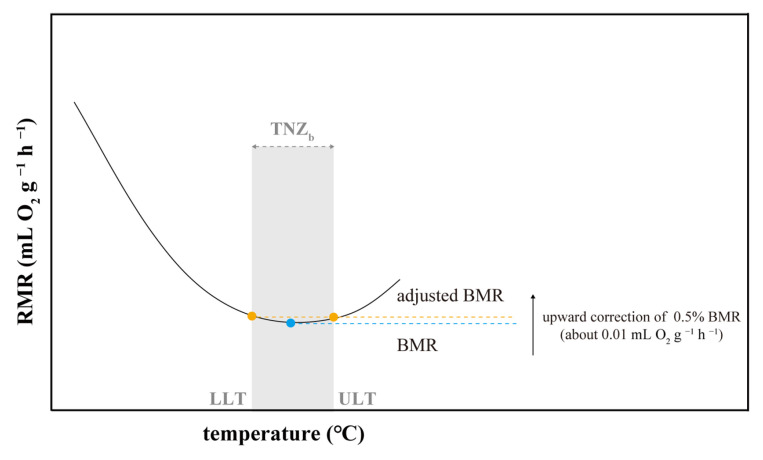
Calculation of the population-specific thermoneutral zone (TNZ) in *A. agrarius*. The black solid line shows an example relationship between the resting metabolic rate (RMR) and ambient temperature (for estimated true relationships in the study populations, see Figure A2). The blue dot indicates the local minimum of the RMR (basal metabolic rate, BMR); the blue dashed line is the corresponding lower asymptote. The orange dashed line is the adjusted BMR (BMR plus a systematic error of 0.01 mL O_2_ g^−1^ h^−1^), and the two orange dots are the lower-limit temperature (LLT) and the upper-limit temperature (ULT). The double-headed arrow indicates the breath of the TNZ (TNZ_b_ = ULT − LLT).

At ambient temperatures ranging from 5 °C to 37.5 °C, the mass-specific resting metabolic rate in *A. agrarius* exhibited a typical U-shaped curve (Figure A1 in Appendix A). For the nine populations of the spatial dataset, the lower-limit temperature (LLT) ranged from 28.64 °C to 30.30 °C, the upper-limit temperature (ULT) ranged from 30.97 °C to 32.21 °C, and the breadth of the thermoneutral zone (TNZ_b_) ranged from 1.34 °C to 2.49 °C (details on these results are provided in Appendix A). Across the spatial temperature gradient, LLT tended to increase (+0.15 °C per +1 °C in AnnMinTemp), and ULT tended to decrease (−0.19 °C per +1 °C in AnnMinTemp), yet neither trend was significant (both *p* > 0.05, Figure 3a). As a result of these (non-significant) trends, TNZ_b_ narrowed significantly with increasing AnnMinTemp (−0.34 °C per +1 °C in AnnMinTemp, *p* < 0.05, Figure 3c). The body size of *A. agrarius*, in turn, decreased significantly with a widening TNZ_b_ (body length: −0.95 ± 0.43 mm °C^−1^; body mass: −0.40 ± 0.19 g °C^−1^; both *p* < 0.05, Figure 3b,d).

### 3.4. Structural Equation Modelling

**Figure 4:**
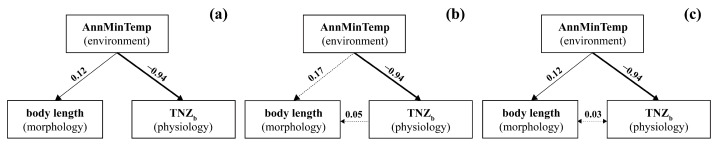
Structural equation models including effects of annual mean minimum temperature (AnnMinTemp), breadth of the thermoneutral zone (TNZ_b_), and body length (body size). The first pathway (**a**) includes only the direct effect of AnnMinTemp on body length and TNZ_b_; the second pathway (**b**) also includes the direct effect of TNZ_b_ on body length; and in the third pathway (**c**) includes the bidirectional effect between TNZ_b_ and body length. Arrows indicate the direction of effects. Solid and dashed lines represent significant (*p* < 0.05) and non-significant (*p* > 0.05) effects, respectively. The thickness of the arrows is scaled to the absolute size of the standardised estimates (numbers next to arrows).

Three structural equation models (SEMs) were constructed to assess the direct and indirect effects of AnnMinTemp and TNZ_b_ on body length. All of them fit the data reasonably well (from left to right in Figure 4, Fisher’s C = 8.36, 7.70, and 7.70; all *p* > 0.05). The third pathway (Figure 4c) had the lowest AIC value (27.70), followed by the first pathway (28.36; Figure 4a) and the second pathway (29.70; Figure 4b). In the third pathway, the direct effects of AnnMinTemp on body length (standardised estimate = 0.12) and TNZ_b_ (standardised estimate = −0.94) were significant (both *p* < 0.05), but the correlation between TNZ_b_ and body length was not significant (standardised estimate = 0.03, *p* > 0.05).

## 4. Discussion

Spatial and temporal changes in the body size of *A. agrarius* were associated with variations in ambient temperature and population-specific TNZs. Body size increased and TNZ narrowed as ambient temperature increased. Across the study area, body size varied along longitude and altitude but not along latitude. However, in contrast to the global scale, where temperature increases with decreasing latitude [49], within our study area, (Guizhou Province) temperature exhibited no clear trend along latitude. Also, in Guizhou Province, altitude increases from east to west [50], which can explain the effect of longitude on body size. Furthermore, from 2013 to 2020, the temporal trends in body size differed between the southern population in Yuqing and the northern population in Gongzhuling. In the southern population, where ambient temperature remained stable over time, body lengths remained stable too; in the northern population, where ambient temperature increased over time, body length also increased (although body mass remained stable at both sites). Our study shows that the extent of body-size change in a rodent species is strongly regulated by the magnitude of change in ambient temperature and population-specific TNZs. These results support our previous hypothesis [4] that the responses of body size to changes in ambient temperature can be attributed to shifts in energy allocation. These shifts, in turn, may be influenced by species-specific TNZs and the extent of warming experienced by different species—or, as shown here, by geographical populations of a single species.

### 4.1. Effects of Ambient Temperature on Body Size

Numerous studies on the effects of climate factors on body-size changes in endotherms have identified temperature as the major predictor of body-size trends (summary in [51]; meta-analysis in [52]). Moreover, several studies on endotherms have shown that individuals are able to alter metabolic rates to some extent and can adapt to variation in energy supply under different ambient temperatures [18,27,53,54,55,56,57,58].

At our nine study sites in Guizhou Province, *A. agrarius* individuals in warmer sites had larger bodies. This trend likely resulted from differential allocation of energy. Over evolutionary time, when *A. agrarius* from the cold ancestral site in northern China colonised our study area [59], individuals whose ancestors had migrated into relatively warm sites needed to expend less energy on maintaining body temperature than individuals whose ancestors had migrated into relatively cold sites and, thus, could allocate more energy to growth [4]. Moreover, because each site had experienced different magnitudes of warming relative to the ancestral site, the extent of body-size change differed among these sites. Notably, while body length increased, body mass remained stable across sites. This decoupling of body length and body mass suggests that these traits may be subject to distinct selective pressures, with body length being more sensitive to thermoregulatory efficiency, while body mass may be stabilised by other factors, such as resource availability or reproductive constraints. The warmer a colonised site, the greater the magnitude of warming experienced at the site relative to the ancestral site. As a consequence, individuals in this site likely spent more time at thermoneutrality and, therefore, grew larger. Therefore, individuals from different geographical populations along a spatial temperature gradient exhibit temperature–size patterns.

Over our study period (2013–2020), ambient temperature significantly rose at the northern site (Gongzhuling), exceeding normal rates of warming but slightly (though non-significantly) declined at the southern site (Yuqing). Accordingly, body length increased in the northern population but remained stable in the southern population. As a result of these distinct trends, the difference in body size between the two populations declined. Notably, over a longer time period (1999–2018), temperature in the south also increased (by about 1 °C), causing the body length of *A. agrarius* to increase as well [4]. These results indicate that noticeable changes in body size require a certain extent of warming. The relative short-term stability of body size in the southern (Yuqing) population was likely a consequence of stable ambient temperature. In addition, local physiological adaptation to warming (i.e., a narrow TNZ) may have restricted the influence of climate warming on body-size change.

### 4.2. Relationship Between the Thermoneutral Zone and Body Size

Our results reveal distinct patterns of body-size response between the northern and southern populations of *A. agrarius*, driven by variations in both temperature trends and thermoneutral zones. When ambient temperature is outside the TNZ of homeothermic individuals, they need more energy to maintain body temperature [60]. This additional investment can affect growth and development and thereby cause variations in a species’ body size [61,62]. Thus, if populations differ in the breadth of their TNZs (TNZ_b_), their body size might respond differently to the same magnitude of warming. Li et al. [4] used this argument to explain the diversity of body-size changes in three rodent species, including our study species, *A. agrarius* (although in their study, the extent of warming differed among the species). In our study, the distinct body-size trends in the northern (Gongzhuling) and the southern (Yuqing) populations—increasing and stable, respectively—can mainly be attributed to the different temperature trends at the northern (increasing) and southern study (slightly but non-significantly decreasing) sites. More generally, in addition to different warming regimes, different body-size trends among populations may also partly result from different TNZ_b_ values. This conclusion is supported by a cross-study comparison. Although we did not measure TNZ_b_ in the northern population, another population in northern China (Changtu County) was reported to have a TNZ_b_ of 4 °C [63]. At this other northern site, the AnnMinTemp over our study period (2013–2020) was 2.61 °C (calculated using data from the ECMWF website: https://cds-beta.climate.copernicus.eu/; accessed on 20 February 2024); at our northern study site (Gongzhuling), it was only 1.76 °C. A broader TNZ_b_ in the northern population suggests that these individuals were more thermally flexible, allowing them to maintain optimal energy expenditure across a wider temperature range and potentially promoting greater body growth in response to rising temperatures. Because the TNZ_b_ in the spatial study increased with decreasing AnnMinTemp, we can assume that the TNZ_b_ of our northern population was at least 4 °C and, thus, larger than that of our southern population (1.64 °C). As a result, assuming equal warming, the northern population needed to invest less energy into maintaining body temperature than the southern population and, therefore, could invest more energy into promoting body growth. Notably, the extent of warming in the northern site was not equal to but larger than that in the southern site, which likely amplified the difference in energy available for body growth between the two populations. Furthermore, although over a longer period (1999–2018), the temperature at the southern study site rose as well [4], the rate of change in body size per 1 °C was lower in the southern population (2.07 ± 0.65 mm °C^−1^) than in the northern population (3.43 ± 1.30 mm °C^−1^). This difference suggests that the comparatively narrow TNZ of the southern population restricted body-size responses to warming.

As has been observed in other species [29,64,65], the *A. agrarius* populations in our study have evolved distinct body sizes along a temperature gradient and developed population-specific TNZs—the longer the body, the narrower the TNZ. However, structural equation modelling did not reveal any causal link between body length and TNZ_b_ (Figure 4). We assume that climate warming affected energy homeostasis less in individuals with an evolved narrow TNZ after their ancestors from the cold ancestral site had colonised warmer sites than in individuals with a steady TNZ equal to that at the ancestral site. Both the narrowing of the TNZ along the temperature gradient and the narrower TNZ in the southern than the northern population likely represent adaptations to the effects of climate warming on body size. Such variation in TNZ_b_ among populations could affect how populations evolve under climate change and ultimately affect their fitness.

**Figure 5:**
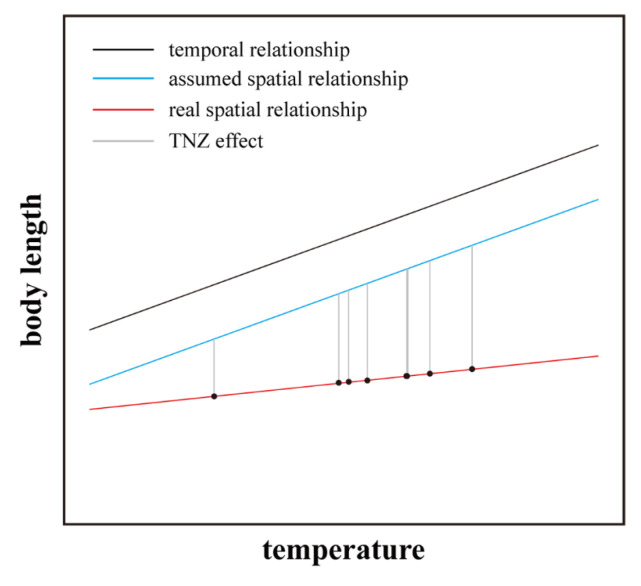
Illustration of how narrowing of the thermoneutral zone (TNZ) in *A. agrarius* with increasing temperature may distinctly restrict the body-length response to temperature change. Originally, *A. agrarius* inhabited a cold (ancestral) site. When individuals colonised warmer areas, they evolved narrower TNZs. The TNZ of the population at the ancestral site, on the other hand, is assumed to remain constant over evolutionary time. The black line shows the relationship between body length and the temporal temperature gradient at the cold ancestral site over evolutionary time. The blue line shows the assumed (i.e., if the TNZ had not narrowed) relationship between body length and the spatial temperature gradient along warm sites in a colonised area. Once individuals have colonised a site in this area, they are assumed to have kept a rather constant TNZ similar to that at the ancestral site. The red line shows the real slope of the relationship between body length and the spatial temperature gradient, where the individuals have evolved a site-specific (or population-specific) TNZ. The grey lines show how population-specific TNZs restrict body-length responses at different site-specific temperatures.

In a previous study [4], we suggested that intraspecific body-size variation driven by temporal temperature variation might be smaller than intraspecific body-size variation driven by spatial temperature variation. Here, however, the opposite was true. While the extent of warming over the eight years at the northern site in Gongzhuling (1.76 °C) was lower than the temperature difference across the nine southern sites in Guizhou Province (3.55 °C), the slope of the relationship between body length and the temporal temperature gradient (3.43 ± 1.30 mm °C^−1^, Gongzhuling) was steeper than the slope of the relationship between body length and the spatial temperature gradient (0.99 ± 0.46 mm °C^−1^, Guizhou Province). This difference may result from the distinct narrowing of the TNZ with increasing temperature, which may distinctly restrict the body-length response to temperature change. If animals in the populations at the colonised warm sites had kept a rather constant TNZ similar to that at the ancestral site, their rate of body-length change along the spatial temperature gradient (blue line in Figure 5) would be similar to the slope of the relationship between body length and the temporal temperature gradient over evolutionary time at the cold ancestral site (black line in Figure 5). However, our results show that *A. agrarius* populations in the warmer sites have evolved narrower TNZs than the population at the cold ancestral site, which has the broadest TNZ [28]. We assume that the breadth of the TNZ at a colonised site was dictated by the difference in temperature between the site and the ancestral site: the warmer a colonised site, the narrower the TNZ of the population at the site and the greater the effect of restricting the body-length response to temperature change (grey lines in Figure 5). As a consequence, the body size in the populations at the warm sites changed only moderately along the spatial temperature gradient (red line in Figure 5). In contrast, because the temperature difference between the northern site and the ancestral site was small, the northern population’s TNZ narrowed less than the southern populations’ TNZ [28]. Therefore, a small increase in temperature over time at the northern site can cause a larger body-size change than the spatial temperature gradient along the warm sites. This difference between temporal and spatial temperature–body size patterns further demonstrates the effects of the TNZ on temperature–size relationships. However, to comprehensively understand the interplay between warming, TNZ, and body size, future studies should examine the effects of not only temperature but also of other environmental variables, such as resource availability and predation pressure, which may interact with thermal constraints in shaping body-size evolution. Moreover, our results indicate that to test Bergmann’s rule, such future studies should include individuals from populations that have experienced a certain magnitude of warming.

## 5. Conclusions

Spatial and temporal body-size patterns in *A. agrarius* deviate from Bergmann’s rule [1]. Our results suggest that the extent of body-size change in a population is strongly affected by two factors: the change in ambient temperature experienced by the population and the population-specific thermoneutral zone (TNZ). As to the first factor, with increasing temperature, either across space or over time, body length increases as well. As to the second factor, individuals with a broad TNZ, typically those in cold areas, are more likely to experience temperatures inside their TNZ. Our results support the hypothesis we postulated previously [4], i.e., that geographical rodent populations have evolved distinct TNZs along environmental temperature gradients and that these population-specific TNZs and the experienced extents of warming likely affect how energy is allocated to body growth and other life processes. However, to thoroughly test the hypothesis, we need more studies covering different species on the interrelations among temporal and spatial temperature gradients, population-specific TNZs, and body size. Future research should prioritise longitudinal studies in regions where rapid warming has occurred, focusing on populations with well-characterised thermophysiological traits to better understand the adaptive responses of body size. Finally, our results help to clarify patterns of climate-driven shifts in body size and thermoregulation. Understanding such patterns is important because body-size changes can have profound ecological and evolutionary consequences, ultimately influencing population survival and ecosystem dynamics.

## Figures and Tables

**Figure A2:**
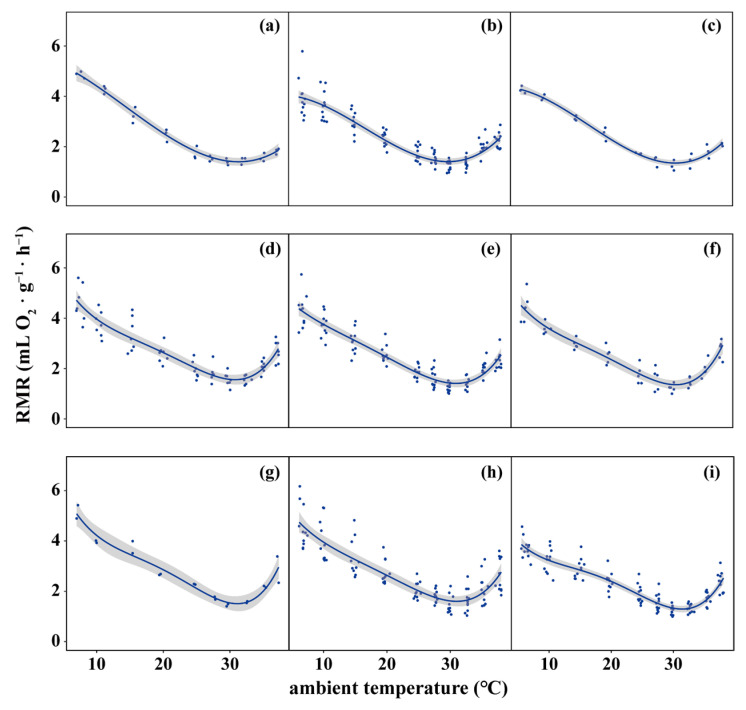
Functional relationship between resting metabolic rate (RMR) and ambient temperature in nine *A. agrarius* populations from distinct sites: (**a**) Dafang, (**b**) Xifeng, (**c**) Weng’an, (**d**) Dejiang, (**e**) Yuqing, (**f**) Kaili, (**g**) Sinan, (**h**) Cengon, and (**i**) Jinping. Dots show the measured values. Curves were fit using the selected best models (Table A1); shaded areas are the 95% confidence intervals.

## Data Availability

The datasets that support the findings of this study are available from the corresponding author upon request.

## Supplementary Materials

The following supporting information can be downloaded at: https://www.mdpi.com/article/10.3390/ani15081112/s1, Figure S1: Distribution (shaded area) and sampling sites for Apodemus agrarius in Guizhou Province and Gongzhuling County, China. Red asterisks and blue dots represent temporal and spatial sampling sites, respectively. The distance between the southern sampling site (21.2177° N) in Yuqing and the northern sampling site (43.7685° N) in Gongzhuling is 2782 km (see inset; Guizhou Province is highlighted in blue). The map of China was derived from the Resource and Environment Data Cloud Platform (http://www.resdc.cn/data.aspx?DATAID=202; accessed on 20 February 2024). The distribution map of A. agrarius was downloaded from the website of the IUCN (http://maps.iucnredlist.org; accessed on 20 February 2024); Table S1: Summary of spatial sampling of A. agrarius; Table S2: Summary of temporal sampling of *A. agrarius*.

## Abbreviations

The following abbreviations are used in this manuscript:

TNZ	Thermoneutral zone
RMR	Resting metabolic rate
LLT	Lower-limit temperature
ULT	Upper-limit temperature
TNZ_b_	The breadth of the TNZ
AnnMinTemp	Annual mean minimum temperature
ECMWF	European Centre for Medium-Range Weather Forecasts
GLMM	Generalised linear mixed-effects model
LM	Linear model
SEM	Structural equation model
AIC	Akaike information criterion

## Appendix A

### Appendix A.1. Methods

#### Appendix A.1.1. Measuring Resting Metabolic Rate (RMR)

We measured RMR in 57 healthy *A. agrarius* adults using indirect calorimetry [35]. Individuals were live-captured in maize fields of nine counties in Guizhou Province, China. For two days leading up to the trials, they were housed individually in plastic cages (L × W × H, 290 mm × 178 mm × 160 mm) in a laboratory in Yuqing County. During this period, food and water (but not nesting substrate) were available. Two hours before the trial, we removed food from the cage. Half an hour before the trial, we measured the animals’ body weight, and put them into a metabolic chamber (volume, 2 L), which was placed inside an incubator. For the first 30 min, the incubator was at room temperature. Then, we started the trial by setting the incubator to one of ten test temperatures: 37.5 °C, 35 °C, 32.5 °C, 30 °C, 27.5 °C, 25 °C, 20 °C, 15 °C, 10 °C, or 5 °C. We tested each animal at all temperatures (from highest to lowest) in ten different trials. A trial at a given temperature lasted 30 min. During each 30 min period, fresh air was pumped into the chamber at a flow rate of 200 mL min^−1^. At each temperature, the corresponding oxygen consumption was recorded automatically at one-minute intervals, using an open-flow respirometry system (SANS Biotechnology Co., Ltd., Beijing, China). RMR was measured as the minimal oxygen consumption during at least 5 min; then, the value was converted to the mass-specific oxygen consumption rate (mL O_2_ g^−1^ h^−1^) [30]. After each trial, we again measured the animal’s body weight.

#### Appendix A.1.2. Calculating the Thermoneutral Zone (TNZ)

Within the thermal tolerance of an endothermic organism, the RMR typically exhibits a U-shaped relationship with temperature. When the temperature increases from the lower-limit temperature (LLT) to the upper-limit temperature (ULT), the RMR first decreases, then reaches a minimum and remains relatively stable for a few °C before finally increasing towards the ULT [66]. The temperature range within which the RMR of a species (or a geographical population) remains low and stable is regarded as the species’ (or the population’s) TNZ [13].

To illustrate the functional relationship between ambient temperature and the mass-specific metabolic rate for each *A. agrarius* population, we constructed different polynomial regression models [67,68]. We started with a first-order polynomial model with RMR as the dependent variable (y) and ambient temperature as the independent variable (x): y = β_0_ + β_1_x. Based on this initial model, we then gradually increased the order: y = β_0_ + β_1_x + β_2X2_ + … + β_n_x_n_. To avoid overfitting, we stopped at the fifth order. To identify the best-fitting curve for each population, we used Akaike’s Information Criterion corrected for small sample sizes (AICc) [69]. We regarded the model with the lowest AICc value as the best model for a population, and used this model to calculate the population’s basal metabolic rate (BMR, the point where RMR is minimal).

Using the gradient descent algorithm [37], a first-order iterative algorithm, we calculated BMR as the local minimum point of the first derivative with respect to temperature (Figure A1) between 27 °C and 37.5 °C. We chose this temperature interval because the lowest LLT in an *A. agrarius* population in a cold zone in Northeast China is 27 °C [63], and the upper limit of thermal tolerance in *A. agrarius* is 37.5 °C [70]. In the iterative algorithm, we set the step-size parameter to 0.01, the accuracy parameter to 10^−6^, and the number of iterations to 10,000—that is, we started the algorithm at 27 °C and calculated the first derivative of the function at each step size. The algorithm terminated when the first derivative at the latest temperature was less than 10^−6^ (the accuracy parameter). The temperature at which this happened coincides with the temperature where the RMR is minimal, which is the basal metabolic rate (BMR) [71]. Because identifying the BMR is affected by an analytic error of 0.5% when, as in our case, nitrogen excretion is neglected [72], we adjusted each population’s BMR upward by 0.5% (about 0.01 mL O_2_ g⁻^1^ h⁻^1^). Then, we computed the RMR values at each 0.01 °C interval using a naive forecasting approach [73]. The lower and upper temperatures at which the RMR of each population equals the adjusted BMR correspond to the population’s ULT and LLT, respectively. Finally, we calculated the breadth of the TNZ (TNZ_b_) as the difference between the ULT and LLT (Figure A1).

All the analyses were conducted in R 4.0 [41]. The polynomial regression model was constructed using the nls2 package [42]. AIC-based model selection was conducted using the AICcmodavg package [47]. Partial derivatives of the model were calculated, and TNZ values were predicted using the stats package [41], the nleqslv package [43], and the numDeriv package [44].

### Appendix A.2. Results

Among the polynomial models evaluated for estimation of the RMR in each of the nine *A. agrarius* populations, the best selected models were either of the third or of the fourth order (Table A1, Figure A2). The population-specific BMR ranged from 1.25 mL O_2_ g^−1^ h^−1^ to 1.71 mL O_2_ g^−1^ h^−1^. Moreover, the LLT ranged from 28.64 °C to 30.30 °C, the ULT ranged from 30.97 °C to 32.21 °C, and the TNZ_b_ ranged from 1.34 °C to 2.49 °C (Table A2).

**Table A1:** Model-selection results for first- to fifth-order polynomial models used to estimate the resting metabolic rate (RMR) as a function of ambient temperature (t) in nine *A. agrarius* populations from distinct sites. For each population, the model with the lowest AICc value (in bold) is regarded as the best model. Akaike weights (*w_i_*) indicate the relative support for each model among the candidate set of models; logLik is the value of the models’ log likelihood.

Site	Model	AICc	*w_i_*	logLik
Dafang	RMR = 4.91 − 1.06 × 10^−1^*t*	52.31	0.00	−22.69
	RMR = 6.92 − 3.41 × 10^−1^*t* + 5.38 × 10^−3^*t*^2^	3.57	0.02	3.01
	RMR **= 5.86 − 1.37 × 10^−1^** *t* ** − 5.13 × 10^−3^** *t* **^2^** ** + 1.58 × 10^−4^** *t* **^3^**	**−3.61**	**0.77**	**8.05**
	RMR = 5.97 − 1.68 × 10^−1^*t* − 2.51 × 10^−3^*t*^2^ + 7.02 × 10^−5^*t*^3^ + 1.00 × 10^−6^*t*^4^	−0.49	0.16	8.07
	RMR = 7.31 − 6.21 × 10^−1^*t* + 5.14 × 10^−2^*t*^2^ − 2.80 × 10^−3^*t*^3^ + 7.13 × 10^−5^*t*^4^ − 6.43 × 10^−7^*t*^5^	2.14	0.04	8.48
Xifeng	RMR = 4.01 − 7.25 × 10^−2^*t*	214.02	0.00	−103.90
	RMR = 5.91 − 2.94 × 10^−1^*t* + 5.04 × 10^−3^*t*^2^	142.71	0.00	−67.17
	RMR **= 3.95 + 7.60 × 10^−2^** *t* ** − 1.38 × 10^−2^** *t* **^2^** ** + 2.80 × 10^−4^** *t* **^3^**	**119.20**	**0.55**	**−54.31**
	RMR = 4.85 − 1.60 × 10^−1^*t* + 5.86 × 10^−3^*t*^2^ − 3.65 × 10^−4^*t*^3^ + 7.27 × 10^−6^*t*^4^	120.24	0.33	−53.71
	RMR = 5.99 − 5.32 × 10^−1^*t* + 4.89 × 10^−2^*t*^2^ − 2.61 × 10^−3^*t*^3^ + 6.11 × 10^−5^*t*^4^ − 4.85 × 10^−7^*t*^5^	122.15	0.13	−53.53
Weng’an	RMR = 4.48 − 9.02 × 10^−2^*t*	51.24	0.00	−22.16
	RMR = 6.41 − 3.14 × 10^−1^*t* + 5.08 × 10^−3^*t*^2^	15.29	0.00	−2.84
	RMR **= 4.12 + 1.17 × 10^−1^***t*** − 1.69 × 10^−2^***t***^2^ + 3.28 × 10^−4^***t***^3^**	**−22.19**	**0.70**	**17.34**
	RMR = 4.73 − 4.17 × 10^−2^*t* − 3.72 × 10^−3^*t* ^2^ − 1.03 × 10^−4^*t*^3^ + 4.86 × 10^−6^*t*^4^	−20.13	0.25	17.89
	RMR = 5.11 − 1.65 × 10^−1^*t* + 1.04 × 10^−2^*t*^2^ − 8.40 × 10^−3^*t*^3^ + 2.26 × 10^−5^*t*^4^ − 1.61 × 10^−7^*t*^5^	−16.78	0.05	17.93
Dejiang	RMR = 4.47 − 7.97 × 10^−2^*t*	148.48	0.00	−71.06
	RMR = 6.60 − 3.28 × 10^−1^*t* + 5.63 × 10^−3^*t*^2^	98.06	0.00	−44.72
	RMR = 4.73 + 2.51 × 10^−2^*t* − 1.23 × 10^−2^*t*^2^ + 2.67 × 10^−4^*t*^3^	87.19	0.17	−38.13
	*R**M**R* **= 7.13 − 5.97 × 10^−1^***t*** + 3.92 × 10^−2^***t***^2^ − 1.42 × 10^−3^***t***^3^ + 1.90 × 10^−5^***t***^4^**	**85.03**	**0.51**	**−35.85**
	RMR =10.14−1.57t + 1.52 × 10^−1^*t*^2^ − 7.29 × 10^−3^*t*^3^ + 1.60 × 10^−4^*t*^4^ − 1.28 × 10^−6^*t*^5^	85.95	0.32	−35.07
Yuqing	RMR = 4.32 − 7.97 × 10^−2^*t*	203.01	0.00	−98.38
	RMR = 6.41 − 3.19 × 10^−1^*t* + 5.40 × 10^−3^*t*^2^	133.99	0.00	−62.78
	RMR = 4.19 + 9.05 × 10^−2^*t* − 1.52 × 10^−2^*t*^2^ + 3.05 × 10^−4^*t*^3^	110.59	0.20	−49.98
	*R**M**R* **= 6.07 − 3.91 × 10^−1^***t*** + 2.45 × 10^−2^***t***^2^ − 9.93 × 10^−4^***t***^3^ + 1.46 × 10^−5^***t***^4^**	**108.75**	**0.49**	**−47.92**
	RMR =8.59−1.20t + 1.16 × 10^−1^*t*^2^ − 5.73 × 10^−3^*t*^3^ + 1.28 × 10^−4^*t*^4^ −1.02 × 10^−6^*t*^5^	109.65	0.31	−47.22
Kaili	RMR = 4.17 − 7.31 × 10^−2^*t*	113.91	0.00	−53.69
	RMR = 6.68 − 3.63 × 10^−1^*t* + 6.54 × 10^−3^*t*^2^	68.83	0.00	−29.97
	RMR = 4.18 + 1.02 × 10^−1^*t*− 1.69 × 10^−2^*t*^2^ + 3.48 × 10^−4^*t*^3^	52.94	0.07	−20.79
	RMR **= 7.34 − 7.05 × 10^−1^** *t* ** + 4.96 × 10^−2^** *t* **^2^** ** − 1.82 × 10^−3^** *t* **^3^** ** + 2.44 × 10^−5^** *t* **^4^**	**48.31**	**0.73**	**−17.18**
	RMR = 8.05 − 9.34 × 10^−1^*t* + 7.56 × 10^−2^*t*^2^ − 3.17 × 10^−3^*t*^3^ + 5.66 × 10^−5^*t*^4^ − 2.89 × 10^−7^*t*^5^	50.95	0.20	−17.14
Sinan	RMR = 4.98 − 9.29 × 10^−2^*t*	49.64	0.00	−21.07
	RMR = 7.63 − 3.95 × 10^−1^*t* + 6.82 × 10^−3^*t*^2^	32.31	0.01	−10.82
	RMR = 4.82 + 1.19 × 10^−1^*t* − 1.90 × 10^−2^*t*^2^ + 3.84 × 10^−4^*t*^3^	27.20	0.19	−6.46
	RMR **= 10.20 − 1.22**t **+ 8.87 × 10^−2^***t***^2^ − 3.09 × 10^−3^***t***^3^ + 3.88 × 10^−5^***t***^4^**	**24.63**	**0.68**	**−3.08**
	RMR = 14.12−2.74t + 2.58 × 10^−1^*t*^2^ − 1.18 × 10^−2^*t*^3^ + 2.45 × 10^−4^*t*^4^ −1.86 × 10^−6^*t*^5^	28.14	0.12	−2.40
Jinping	RMR $=3.66-6.22\;\times \;10^{-2}t$	167.41	0.00	−80.58
	RMR $=5.32-2.56\;\times \;10^{-1}t$ + 4.41 × 10^−3^t^2^	100.3	0.00	−45.94
	RMR $=3.11+1.66\;\times \;10^{-1}t$ $\;-\;1.72\;\times \;10^{-2}t$ ^2^ $\;+\;3.25\;\times \;10^{-4}t$ ^3^	55.69	0.03	−22.53
	RMR ** = 4.89 − 3.02 × 10^−1^** t ** + 2.20 × 10^−2^** t **^2^** ** − 9.73 × 10^−4^** t **^3^** ** + 1.47 × 10^−5^** t **^4^**	**50.18**	**0.53**	**−18.64**
	RMR $=6.77-9.26\;\times \;10^{-1}t$ $\;+\;9.49\;\times \;10^{-2}t$ ^2^ $\;-\;4.81\;\times \;10^{-3}t$ ^3^ $\;+\;1.08\;\times \;10^{-4}t$ ^4^ $\;-\;8.46\;\times \;10^{-7}t$ ^5^	50.59	0.43	−17.69
Cengon	RMR = 4.47 − 7.94 × 10^−2^*t*	192.29	0.00	−93.03
	RMR = 6.70 − 3.38 × 10^−1^*t* + 5.87 × 10^−3^*t*^2^	141.47	0.00	−66.54
	RMR = 4.73 + 3.03 × 10^−2^*t*^−1^ − 1.28 × 10^−2^*t*^2^ + 2.78 × 10^−4^*t*^3^	108.35	0.00	−48.89
	RMR **= 7.23 − 6.14 × 10^−1^** *t* ** + 4.05 × 10^−2^** *t* **^2^** ** − 1.46 × 10^−3^** *t* **^3^** ** + 1.96 × 10^−5^** *t* **^4^**	**95.14**	**0.63**	**−41.16**
	RMR =10.86−1.79t+ 1.75 × 10^−1^*t*^1^ − 8.46 × 10^−3^*t*^3^ + 1.87 × 10^−4^*t*^4^ − 1.51 × 10^−6^*t*^5^	96.18	0.37	−40.54

**Table A2:** Thermoneutral zones (TNZs) of nine *A. agrarius* populations from distinct sites. ULT, LLT, and TNZ_b_ are the upper-limit temperature, the lower-limit temperature, and the breadth of the TNZ, respectively. The TNZ extends from the LLT to the ULT.

Site	Sample Size	BMR (mL O_2_ g ^−1^ h ^−1^)	ULT (°C)	LLT (°C)	TNZ_b_ (°C)
Dafang	3	1.39	32.21	29.72	2.49
Xifeng	9	1.37	30.97	28.64	2.33
Wengan	3	1.27	31.37	29.50	1.87
Dejiang	7	1.54	31.88	29.83	2.05
Yuqing	8	1.39	31.65	30.01	1.64
Kaili	5	1.45	31.00	29.40	1.60
Sinan	2	1.40	31.88	30.30	1.58
Jinping	10	1.25	31.39	30.05	1.34
Cengon	10	1.71	31.09	29.47	1.62
Total/Average	57	1.42	31.49	29.66	1.84
