# Supplementary material for: Body-Size Change in a Rodent Is Affected by Environmental Warming and Population-Specific Thermoneutral Zone
Source: Animals (Basel). 2025 Apr 11;15(8):1112. doi: 10.3390/ani15081112 (PMC12024339; doi:10.3390/ani15081112)
Supplement: Supplementary file 1 [file animals-15-01112-s001.zip › SuppInfo.pdf]

Supporting Information

# Body-size change in a rodent is affected by environmental warming and population-specific thermoneutral zone

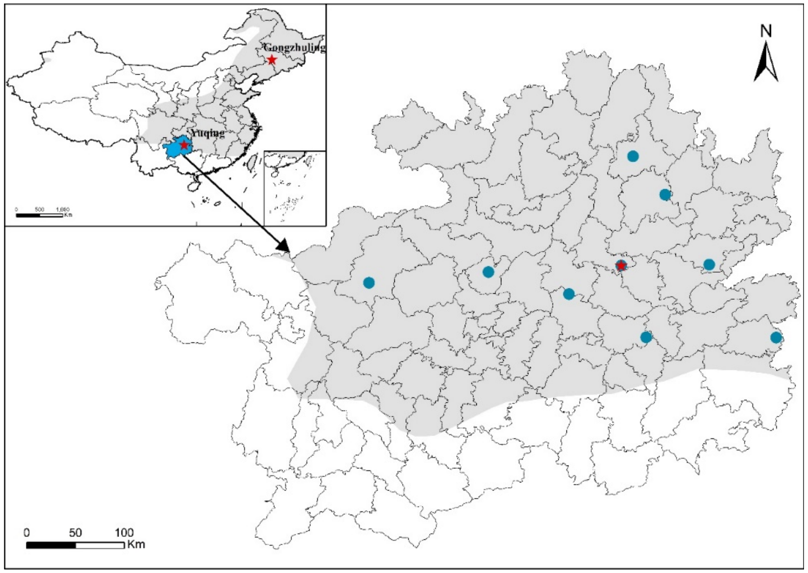


**Figure S1** Distribution (shaded area) and sampling sites for *Apodemus agrarius* in Guizhou Province and Gongzhuling County, China. Red asterisks and blue dots represent temporal and spatial sampling sites, respectively. The distance between the southern sampling site (21.2177° N)

in Yuqing and the northern sampling site (43.7685° N) in Gongzhuling is 2,782 km (see inset; Guizhou Province is highlighted in blue). The map of China was derived from the Resource and Environment Data Cloud Platform (http://www.resdc.cn/data.aspx?DATAID=202). The distribution map of *A. agrarius* was downloaded from the website of the IUCN (http://maps.iucnredlist.org).

**Table S1** Summary of spatial sampling of *A. agrarius*

| Sampling site | Sampling year | Longitude (°E) | Latitude (°N) | Altitude (m) | Number of non-pregnant females with body-size data per stage | | | | | | Number of rodents used for body-size analysis, including Sub-adult, Adult I, and Adult II stages only | | | Number of rodents captured in 2023 for TNZ analysis | | |
|---|---|---|---|---|---|---|---|---|---|---|---|---|---|---|---|---|
| | | | | | Totol | Juvenile | Sub-adult | Adult I | Adult II | Old | Total | Male | Female | Total | Male | Female |
| Cengon | 2022-2023 | 108.7150 | 27.2429 | 387.53 | 27 | 0 | 4 | 8 | 10 | 5 | 22 | 6 | 16 | 10 | 6 | 4 |
| Dafang | 2022-2022 | 105.5800 | 27.0748 | 1509.00 | 19 | 0 | 3 | 11 | 5 | 0 | 19 | 11 | 8 | 3 | 0 | 3 |
| Dejiang | 2023 | 108.0121 | 28.2399 | 635.90 | 16 | 2 | 7 | 5 | 2 | 0 | 14 | 5 | 9 | 7 | 4 | 3 |
| Jinping | 2022-2023 | 109.3322 | 26.5710 | 359.40 | 25 | 0 | 9 | 12 | 4 | 0 | 25 | 15 | 10 | 10 | 5 | 5 |
| Kaili | 2022-2023 | 108.1340 | 26.5725 | 672.50 | 19 | 0 | 3 | 12 | 4 | 0 | 19 | 11 | 8 | 5 | 4 | 1 |
| Sinan | 2022-2023 | 108.3089 | 27.8868 | 545.96 | 16 | 1 | 5 | 6 | 4 | 0 | 15 | 7 | 8 | 2 | 2 | 0 |
| Wengan | 2022-2023 | 107.5688 | 26.9753 | 1208.12 | 12 | 0 | 5 | 1 | 6 | 0 | 12 | 9 | 3 | 3 | 1 | 2 |

| | | | | | | | | | | | | | | | |
|---|---|---|---|---|---|---|---|---|---|---|---|---|---|---|---|
| Xifeng | 2022-2023 | 106.5921 | 27.1653 | 1195.20 | 18 | 1 | 4 | 4 | 7 | 2 | 15 | 6 | 9 | 9 | 6 | 3 |
| Yuqing | 2022-2023 | 107.8875 | 27.2177 | 558.40 | 28 | 1 | 9 | 6 | 7 | 5 | 22 | 8 | 14 | 8 | 5 | 3 |
| Total | n/a | n/a | n/a | n/a | 180 | 5 | 49 | 65 | 49 | 12 | 163 | 78 | 85 | 57 | 33 | 24 |

**Table S2** Summary of temporal sampling of *A. agrarius*

| Sampling site | Sampling year | Number of rodents used for body-size analysis, including Juvenile, Sub-adult, Adult I, Adult II, and Old stages | | | Number of rodents with body-size data per stage | | | | | Number of rodents used for body-size analysis, including Sub-adult, Adult I, and Adult II stages only |
|---|---|---|---|---|---|---|---|---|---|---|
| | | Total | Male | Female | Juvenile | Sub-adult | Adult I | Adult II | Old | Total |
| Yuqing | 2013 | 165 | 64 | 101 | 1 | 53 | 43 | 49 | 19 | 145 |
| | 2014 | 260 | 102 | 158 | 32 | 95 | 63 | 56 | 14 | 214 |
| | 2015 | 299 | 107 | 192 | 30 | 97 | 82 | 69 | 21 | 248 |
| | 2016 | 289 | 118 | 171 | 32 | 93 | 78 | 73 | 13 | 244 |
| | 2017 | 249 | 106 | 143 | 5 | 88 | 64 | 76 | 16 | 228 |
| | 2018 | 161 | 67 | 94 | 12 | 71 | 53 | 20 | 5 | 144 |
| | 2019 | 255 | 124 | 131 | 26 | 92 | 64 | 62 | 11 | 218 |
| | 2020 | 167 | 63 | 104 | 7 | 31 | 62 | 54 | 13 | 147 |

| | | | | | | | | | | |
|---|---|---|---|---|---|---|---|---|---|---|
| | **Total** | **1845** | **751** | **1094** | **145** | **620** | **509** | **459** | **112** | **1588** |
| | 2013 | 111 | 70 | 41 | 35 | 43 | 22 | 8 | 3 | 73 |
| | 2014 | 137 | 87 | 50 | 52 | 47 | 25 | 13 | 0 | 85 |
| | 2015 | n/a | n/a | n/a | n/a | n/a | n/a | n/a | n/a | n/a |
| | 2016 | n/a | n/a | n/a | n/a | n/a | n/a | n/a | n/a | n/a |
| Gongzhuling (for body length analysis) | 2017 | n/a | n/a | n/a | n/a | n/a | n/a | n/a | n/a | n/a |
| | 2018 | 32 | 21 | 11 | 18 | 5 | 8 | 0 | 1 | 13 |
| | 2019 | 98 | 53 | 45 | 33 | 36 | 17 | 10 | 2 | 63 |
| | 2020 | 119 | 89 | 30 | 45 | 46 | 21 | 6 | 1 | 73 |
| | **Total** | **497** | **320** | **177** | **183** | **177** | **93** | **37** | **7** | **307** |
| | 2013 | 111 | 70 | 41 | 35 | 43 | 22 | 8 | 3 | 73 |
| | 2014 | 137 | 87 | 50 | 52 | 47 | 25 | 13 | 0 | 85 |
| | 2015 | 110 | 87 | 23 | 42 | 39 | 18 | 10 | 1 | 67 |
| | 2016 | 134 | 99 | 35 | 32 | 38 | 36 | 26 | 2 | 100 |
| Gongzhuling (for body mass analysis) | 2017 | 111 | 77 | 34 | 41 | 39 | 21 | 8 | 2 | 68 |
| | 2018 | 34 | 22 | 12 | 19 | 5 | 8 | 1 | 1 | 14 |
| | 2019 | 103 | 56 | 47 | 35 | 36 | 20 | 10 | 2 | 66 |
| | 2020 | 129 | 98 | 31 | 49 | 47 | 24 | 8 | 1 | 79 |
| | **Total** | **869** | **596** | **273** | **305** | **294** | **174** | **84** | **12** | **552** |
